# Rates of COVID-19 Among Residents and Staff Members in Nursing Homes — United States, May 25–November 22, 2020

**DOI:** 10.15585/mmwr.mm7002e2

**Published:** 2021-01-15

**Authors:** Suparna Bagchi, Josephine Mak, Qunna Li, Edward Sheriff, Elisabeth Mungai, Angela Anttila, Minn Minn Soe, Jonathan R. Edwards, Andrea L. Benin, Daniel A. Pollock, Evan Shulman, Shari Ling, Jean Moody-Williams, Lee A. Fleisher, Arjun Srinivasan, Jeneita M. Bell

**Affiliations:** ^1^CDC COVID-19 Response Team; ^2^Centers for Medicare & Medicaid Services.

During the beginning of the coronavirus disease 2019 (COVID-19) pandemic, nursing homes were identified as congregate settings at high risk for outbreaks of COVID-19 (*1*,*2*). Their residents also are at higher risk than the general population for morbidity and mortality associated with infection with SARS-CoV-2, the virus that causes COVID-19, in light of the association of severe outcomes with older age and certain underlying medical conditions (*1*,*3*). CDC’s National Healthcare Safety Network (NHSN) launched nationwide, facility-level COVID-19 nursing home surveillance on April 26, 2020. A federal mandate issued by the Centers for Medicare & Medicaid Services (CMS), required nursing homes to commence enrollment and routine reporting of COVID-19 cases among residents and staff members by May 25, 2020. This report uses the NHSN nursing home COVID-19 data reported during May 25–November 22, 2020, to describe COVID-19 rates among nursing home residents and staff members and compares these with rates in surrounding communities by corresponding U.S. Department of Health and Human Services (HHS) region.* COVID-19 cases among nursing home residents increased during June and July 2020, reaching 11.5 cases per 1,000 resident-weeks (calculated as the total number of occupied beds on the day that weekly data were reported) (week of July 26). By mid-September, rates had declined to 6.3 per 1,000 resident-weeks (week of September 13) before increasing again, reaching 23.2 cases per 1,000 resident-weeks by late November (week of November 22). COVID-19 cases among nursing home staff members also increased during June and July (week of July 26 = 10.9 cases per 1,000 resident-weeks) before declining during August–September (week of September 13 = 6.3 per 1,000 resident-weeks); rates increased by late November (week of November 22 = 21.3 cases per 1,000 resident-weeks). Rates of COVID-19 in the surrounding communities followed similar trends. Increases in community rates might be associated with increases in nursing home COVID-19 incidence, and nursing home mitigation strategies need to include a comprehensive plan to monitor local SARS-CoV-2 transmission and minimize high-risk exposures within facilities.

On May 25, 2020, CMS-certified nursing homes began reporting data to NHSN in response to a federal mandate (*4*). This reporting included data on the number of beds occupied and the number of COVID-19 cases among residents and staff members confirmed by antigen tests or laboratory-based viral nucleic acid test results (*5*). Nursing home staff members and facility personnel comprise all persons working or volunteering in the facility, including contractors, temporary staff members, resident caregivers, and staff members who might work at multiple facilities (*5*). Data on COVID-19 cases among residents and staff members reported during May 25–November 22, 2020 were analyzed for nursing homes in all U.S. states, the District of Columbia, Guam, and Puerto Rico. Facilities are expected to enter incident COVID-19 case counts on residents and staff members weekly. Facilities were excluded from the analysis for specific weeks if data on cases, occupied beds, or staffing were not reported. Data quality checks indicated that in some cases, facilities might have misinterpreted instructions and that cumulative case counts, rather than weekly case counts, were being entered. Based on the pattern of data entry, if it appeared that cumulative data were entered consecutively, data field values were reassigned to a weekly incident value. Outlier data points were derived using the distribution of facility-level resident and staff member case counts reported on a single collection date among reporting nursing homes over the entire cohort during the data collection period, and any value above the 99.9th percentile (i.e., >55 cases for residents and >37 cases for staff members) was truncated to the corresponding cut-point value. Case count data were aggregated weekly, and resident-weeks were calculated as the total number of occupied beds on the day data were reported. Because data on number of staff members employed is not collected, the proxy denominator of resident-weeks was used as a closest best estimate of the at-risk denominator for staff members. Weekly incidence was calculated for the weekly aggregated data at the end of each calendar week. Cases per 1,000 resident-week were calculated for residents and staff members using the number of COVID-19 cases reported in a week over the corresponding 1,000 resident-weeks. Community COVID-19 rates per 100,000 population were calculated for each of the ten HHS regions as the total number of cases reported in a week over the region’s estimated population, using data available at USAFacts.org (*6*). Calculations of cases per 100,000 population in Region 2 excluded cases reported from Puerto Rico and in HHS Region 9 excluded cases reported from Guam. Rates among residents and staff members and in the surrounding community were compared by HHS region. Analyses were conducted using SAS software (version 9.4; SAS Institute). This activity was reviewed by CDC and was conducted consistent with applicable federal law and CDC policy.^†^

Among 15,404 nursing homes, 15,342 (99.6%) were included in the analysis. Overall, 13,185 (86%) nursing homes had ≥50 beds, 10,750 (70.1%) were for-profit, and 14,349 (93.5%) had dual Medicare and Medicaid certification (Table). Most nursing homes (8,688; 62.2%) were in HHS Regions 4, 5, 6, and 7.

**TABLE:** Characteristics of nursing homes reporting COVID-19 to the National Healthcare Safety Network (N = 15,342) — United States, May 25–November 22, 2020

Characteristic	No. (%)
Facility bed size*	
<50	2,126 (13.9)
50–99	5,533 (36.1)
100–199	6,764 (44.1)
>199	888 (5.8)
Unknown^†^	31 (0.2)
Facility ownership*	
Not-for-profit	3,678 (24.0)
For-profit	10,750 (70.1)
Government	883 (5.8)
Unknown^†^	31 (0.2)
Certification*	
Dual Medicare and Medicaid	14,349 (93.5)
Medicare only	652 (4.2)
Medicaid only	310 (2.0)
Unknown^†^	31 (0.2)
**HHS regions** ^§^	
Region 1	836 (6.0)
Region 2	909 (6.5)
Region 3	1,225 (8.8)
Region 4	2,329 (16.7)
Region 5	3,108 (22.2)
Region 6	1,880 (13.5)
Region 7	1,371 (9.8)
Region 8	567 (4.1)
Region 9	1,334 (9.5)
Region 10	414 (3.0)

**Abbreviations:** COVID-19 = coronavirus disease 2019, HHS = U.S. Department of Health and Human Services.

* Data source: https://data.medicare.gov/Nursing-Home-Compare/Provider-Info/4pq5-n9py/data. Unknown category includes nursing homes where the information is not available.

^†^ Unknown represents facilities without information on bed size, facility ownership, and certification

^§^ *Region 1:* Connecticut, Maine, Massachusetts, New Hampshire, Rhode Island, and Vermont; *Region 2:* New Jersey, New York, and Puerto Rico; *Region 3:* Delaware, District of Columbia, Maryland, Pennsylvania, Virginia, and West Virginia; *Region 4:* Alabama, Florida, Georgia, North Carolina, Kentucky, Mississippi, South Carolina, and Tennessee; *Region 5:* Illinois, Indiana, Michigan, Minnesota, Ohio, and Wisconsin; *Region 6:* Arkansas, Louisiana, New Mexico, Oklahoma, and Texas; *Region 7:* Iowa, Kansas, Missouri, and Nebraska; *Region 8:* Colorado, Montana, North Dakota, South Dakota, Utah, and Wyoming; *Region 9:* Arizona, California, Hawaii, Nevada, Guam; *Region 10:* Alaska, Idaho, Oregon, and Washington.

During May 25–November 22, nursing homes reported 572,135 cases to NHSN, 296,762 (51.8%) of which occurred among residents and 275,373 (48.2%) among staff members. Among residents, cases per 1,000 resident-weeks increased during June and July, reaching 11.5 cases per 1,000 resident-weeks (week of July 26), and decreased during August–September (week of September 13 incidence = 6.3 per 1,000 resident-weeks). In November, rates increased again, reaching 23.2 cases per 1,000 resident-weeks (week of November 22) (Figure). Among staff members, cases per 1,000 resident-weeks also increased during June and July, reaching 10.9 cases per 1,000 resident-weeks (week of July 26); incidence then decreased during August and September (week of September 13, 2020 incidence = 6.3 per 1,000 resident-weeks). Incidence among staff members also increased in November, reaching 21.3 cases per 1,000 resident-weeks during the week of November 22 (Figure). Although incidence among residents (10.5 cases per 1,000 resident-weeks) was higher than that among staff members (8.9 per 1,000 resident-weeks) on May 31, during increases in July and November incidence among staff members closely matched that among residents, and trends were similar.

**FIGURE:**
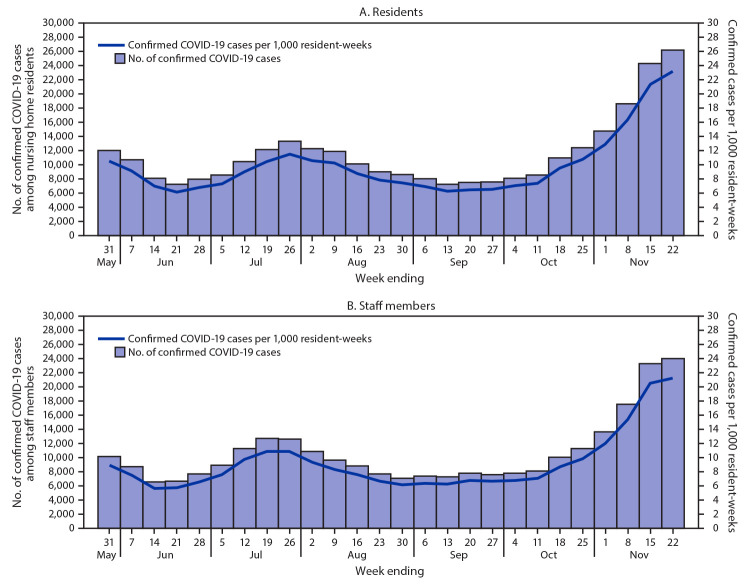
COVID-19 cases* per 1,000 resident-weeks^†^ among nursing home residents (A) and staff members (B) — United States, May 25–November 22, 2020 **Abbreviation:** COVID-19 = coronavirus disease 2019. * Confirmed COVID-19 cases were diagnosed by a positive SARS-CoV-2 viral nucleic acid or antigen test. ^†^ Resident-weeks were calculated as the total number of occupied beds on the day data were reported.

Nursing homes in HHS Regions 1 and 2 reported peak incidences of >10.0 cases per 1,000 resident-weeks among residents and staff members during May or June before rates subsequently declined to <6.0 cases per 1,000 resident-weeks during June–October (Supplementary Figure, https://stacks.cdc.gov/view/cdc/99807). During the July peak, rates among residents and staff members in HHS Regions 4, 6, and 9 ranged from 14 to 24 cases per 1,000 resident-weeks. In HHS Regions 5, 7, and 8, rates ranged from 2.5 to 15 cases per 1,000 resident-weeks during August–September and increased again in November, ranging from 32 to 44 cases per 1,000 resident-weeks during the week of November 22.

During May, population-level COVID-19 rates across the HHS regions ranged from 17 to 67 cases per 100,000 population, and during the July peak, increased to >178 cases per 100,000 in HHS Regions 4, 6, and 9. Rates declined in all HHS regions during August–September and began increasing again in October, with rates in HHS Region 5, 7, and 8 exceeding 615 cases per 100,000 during the week of November 22. For each HHS region, trends in nursing home incidence among residents and staff members were similar to population trends in the surrounding community.

## Discussion

There has been a substantial incidence of COVID-19 among nursing home residents and staff since May 2020. Rates of COVID-19 among residents and staff members in nursing homes fluctuated during weeks ending May 31–November 22, with regional and temporal variability; however, trends resembled those in the surrounding communities. These data suggest that increases in community rates might be associated with increases in nursing home COVID-19 incidence and that nursing home mitigation strategies need to include a comprehensive plan to monitor local SARS-CoV-2 transmission and minimize high-risk exposures within facilities. Increased COVID-19 incidence in communities with nursing homes increases the risk for introduction of SARS-CoV-2 by staff members. In Minnesota, ≥34% of high-risk exposures among health care staff members involved nonpatient contacts, including household and social contacts, indicating potential lapses in adherence to mask use and social distancing recommendations during social interactions (*7*). Addressing health care safety gaps calls for educating staff members about the risk for community exposure, encouraging consistent use of CDC guidance^§^ in all settings, as well as ensuring adequate access and availability of personal protective equipment (*8*). In addition, nursing home adherence to the CMS requirement to conduct routine testing among all staff members and isolate newly admitted or readmitted residents with an unknown COVID-19 status can reduce the risk for SARS-CoV-2 introduction into nursing homes (*9*).

The findings in this report are subject to at least four limitations. First, nursing homes reported aggregate weekly data to NHSN, preventing patient-level analysis. Second, reported data were not validated, and trends among nursing homes excluded because of missing data might have differed. Third, the sources of introduction and direction of transmission between residents and staff members could not be determined. Finally, these results might not be generalizable to residents and staff members of other long-term care facilities, such as those for the developmentally disabled and assisted living facilities because this analysis was restricted to nursing homes reporting COVID-19 data weekly, as required by CMS.

Nursing homes are high-risk, congregate settings that require a comprehensive infection prevention and control strategy to reduce SARS-CoV-2 entry into the facility and mitigate transmission to prevent severe outcomes. CDC’s nursing home guidance provides tiered recommendations for different phases of a COVID-19 response and should be implemented in addition to CMS regulatory requirements (*9*). Prioritization of nursing home residents and staff members for SARS-CoV-2 vaccination, as recommended by the Advisory Committee on Immunization Practices, is an additional strategy to assist mitigation (*10*). Guidance and federal requirements could be further improved through assessing factors associated with the incidence of COVID-19 among nursing home staff members and residents, including factors associated with community-acquired infections leading to transmission within nursing homes.

**Summary**

**What is already known about this topic?**

In the United States, COVID-19 among older adults living in nursing homes is associated with higher rates of severe illness and death.

**What is added by this report?**

Rates of COVID-19 among nursing home residents and staff members increased during June and July 2020, and again in November. Trends in reported COVID-19 cases among nursing home residents and staff members were similar to trends in incidence of COVID-19 in surrounding communities.

**What are the implications for public health practice?**

Increases in community rates might be associated with increases in nursing home COVID-19 incidence, and nursing home mitigation strategies need to include a comprehensive plan to monitor local SARS-CoV-2 transmission and minimize high-risk exposures within facilities.

## Footnotes

* https://www.hhs.gov/about/agencies/iea/regional-offices/index.html.

† 45 C.F.R. part 46.102(l)(2), 21 C.F.R. part 56; 42 U.S.C. Sect. 241(d); 5 U.S.C. Sect. 552a; 44 U.S.C. Sect. 3501 et seq.

§ https://www.cdc.gov/coronavirus/2019-ncov/prevent-getting-sick/prevention.html.

## References

[1] McMichael TM, Clark S, Pogosjans S, ; Public Health–Seattle & King County; EvergreenHealth; CDC COVID-19 Investigation Team. COVID-19 in a long-term care facility—King County, Washington, February 27–March 9, 2020. MMWR Morb Mortal Wkly Rep 2020;69:339–42. 10.15585/mmwr.mm6912e1 32214083 PMC7725515

[2] Telford CT, Onwubiko U, Holland DP, Preventing COVID-19 outbreaks in long-term care facilities through preemptive testing of residents and staff members—Fulton County, Georgia, March–May 2020. MMWR Morb Mortal Wkly Rep 2020;69:1296–9. 10.15585/mmwr.mm6937a4 32941413 PMC7498169

[3] Bialek S, Boundy E, Bowen V, ; CDC COVID-19 Response Team. Severe outcomes among patients with coronavirus disease 2019 (COVID-19)—United States, February 12–March 16, 2020. MMWR Morb Mortal Wkly Rep 2020;69:343–6. 10.15585/mmwr.mm6912e2 32214079 PMC7725513

[4] Centers for Medicare & Medicaid Services. Interim final rule updating requirements for notification of confirmed and suspected COVID-19 cases among residents and staff in nursing homes. Baltimore, MD: US Department of Health and Human Services, Centers for Medicare & Medicaid Services; 2020. https://www.cms.gov/files/document/qso-20-29-nh.pdf

[5] CDC. Instructions for completion of the COVID-19 long-term care facility (LTCF) staff and personnel impact form (CDC 57.145). Atlanta, GA: US Department of Health and Human Services, CDC; 2020. https://www.cdc.gov/nhsn/pdfs/covid19/ltcf/57.145-toi-508.pdf

[6] USAFacts. Detailed methodology and sources: COVID-19 data. Bellevue, WA: USAFacts; 2020. https://usafacts.org/articles/detailed-methodology-covid-19-data/

[7] Fell A, Beaudoin A, D’Heilly P, ; Minnesota Department of Health COVID-19 HCW Monitoring Response Team; Minnesota Department of Health COVID-19 Response Task Force. SARS-CoV-2 exposure and infection among health care personnel—Minnesota, March 6–July 11, 2020. MMWR Morb Mortal Wkly Rep 2020;69:1605–10. 10.15585/mmwr.mm6943a5 33119557 PMC7641003

[8] Honein MA, Christie A, Rose DA, ; CDC COVID-19 Response Team. Summary of guidance for public health strategies to address high levels of community transmission of SARS-CoV-2 and related deaths, December 2020. MMWR Morb Mortal Wkly Rep 2020;69:1860–7. 10.15585/mmwr.mm6949e2 33301434 PMC7737690

[9] Centers for Medicare & Medicaid Services. Interim final rule (IFC). CMS-3401-IFC, Additional policy and regulatory revisions in response to the COVID-19 public health emergency related to long-term care (LTC) facility testing requirements and revised COVID-19 focused survey tool. Baltimore, MD: US Department of Health and Human Services, Centers for Medicare & Medicaid Services; 2020. https://www.cms.gov/files/document/qso-20-38-nh.pdf

[10] Dooling K, McClung N, Chamberland M, The Advisory Committee on Immunization Practices’ interim recommendation for allocating initial supplies of COVID-19 vaccine—United States, 2020. MMWR Morb Mortal Wkly Rep 2020;69:1857–9. 10.15585/mmwr.mm6949e1 33301429 PMC7737687

